# Assessment of AflatoxinM1 Contamination in UHT Flavored Milk Samples in Karaj, Iran

**Published:** 2016

**Authors:** Zohreh Mashak, Hadi Jafari Sohi, Ali Heshmati, Amir Sasan Mozaffari Nejad

**Affiliations:** a *Department of Food Hygiene, Karaj Branch, Islamic Azad University, Karaj, Iran. *; b *bNutrition Health Research Center, Hamadan University of Medical Sciences, Hamadan, Iran.*; c *cMolecular Research Center, Hamadan University of Medical Sciences, Hamadan, Iran. *

**Keywords:** Aflatoxin M_1_, Ultra high temperature milk, High performance liquid chromatography, Iran

## Abstract

This study was carried out to detect the presence of aflatoxin M_1_ (AFM_1_) in 30 UHT flavored milk samples in Karaj, Alborz province, Iran. High-performance liquid chromatography (HPLC) was applied to analyze AFM_1_ in the samples. The results showed that aflatoxin M_1 _was detected in all the UHT flavored milk samples, the AFM_1_ concentration ranged from 0.015 to 0.14 µg/L. Also, 10 samples (33.3%) were contaminated with more than 0.05 µg/L of European Union regulations for aflatoxin M_1_. Wherease, according to the proposed Iranian national standard and FDA (0.5 µg/L), none of the samples has not been contaminated more than the maximum AFM_1 _concentrations threshold. This is the first report discovering the fact that UHT flavored milk is an important contributor to the dietary intake of AFM_1_ in Iran.

## Introduction

Mycotoxins are toxic secondary metabolites produced in several agricultural commodities and foods, during harvesting, storage and processing, also, under suitable conditions of temperature and humidity (1-3). Aflatoxins are the most generally known groups of mycotoxins worldwide, which are produced by the current fungi *Aspergillus flavus, A. parasiticus, A. pseudotamarii, A. bombycis, A. ochraceoroseus*and* A. Nomius *(1 ,4). Major kinds of aflatoxins are B_1_, B_2_, G_1_, G_2_, M_1_ and M_2_. They are highly toxic, teratogenic, estrogenic, mutagenic, tremorogenic, immunosuppressive and carcinogenic compounds which have been implicated as causative agent in human hepatic and extrahepatic carcinogenesis (1, 5-6). Among them, aflatoxin B_1_ (AFB_1_) is the most potent reason of human carcinogen; therefore, the International Agency for Research on Cancer (IARC) has classified AFB_1_ and AFM_1 _into the primary and secondary groups of carcinogenic compounds, respectively (3, 5, 7). When animals eat foodstuffs containing AFB_1_, these toxins will be metabolized and extracted as AFM_1_ in milk (1).

Flavored milk is one of the dairy products facing an increased consumption in Iran, also it is most popular among children and infant. Flavored milk is cow’s milk with added sugar, colourings and artificial or natural flavourings. The current available flavours are chocolate, banana, strawberry and vanilla. It contains nine essential nutrients such as calcium, potassium, phosphorus, protein, vitamins A, D and B12, riboflavin and niacin. 

Milk and dairy product are a concern for human health, particularly to children worldwide (8). Hence, several countries have set legal regulations to control levels of aflatoxin M_1_ in milk and dairy products which vary from one country to another (1, 5). Kamkar *et al.* (1) reported that aflatoxin M_1_ is resistant to heat treatments such as pasteurization, sterilization (UHT techniques), autoclaving, and other processes like freezing, fermentation and cold storage. Several analytical and immunological analytical techniques such as TLC, ELISA and HPLC are available for determination of AFM_1_ in milk and dairy products. HPLC methods are very costly, precise, selective, simple and sensitive techniques for aflatoxins analysis (1, 9). 

The consumption of flavored milk is common among children and school children. Several studies about aflatoxin occurrence in milk and dairy products have been reported in Iran (1). Hence, the aim of this study was to investigate the first prevalence of AFM_1_ in UHT flavored milk samples in Karaj, Alborz province, Iran. 

**Table 1:** Occurrence and level of AFM_1_ in UHT flavored milk in Iran

**Analysed samples**	**Positive samples(%)**	**Frequency distribution (%) (µg/L)**					**Range (µg/L)** a	**Average** b **(µg/L)**
		<LOQ	LOQ- 0.05	0.05- 0.1	0.1- 0.5	> 0.5		
30	30 (100)	5 (16.7 %)	15 (50 %)	4 (13.3 %)	6 (20 %)	0	<LOQ – 0.14	0.079 ± 0.032

a Min – Max.

b Mean of positive samples ± SD.

**Table 2:** Occurrence and levels of AFM_1_ in UHT milks reported in previous studies

**Country**	**Sample**	**Positive (%)**	**Range (µg/L)**	**No> 0.05 (µg/L) (%)**	**Method**	**Reference**
Iran	109	62.3	0.0464-0.01	17.4	ELISA	Fallah (8)
Iran	210	55.2	<0.002-0.005	33.3	ELISA	Heshmati and Milani (9)
Turkey	129	58.1	0–0.54364	47	ELISA	Unusan (14)
Turkey	100	67	0.01-0.63	31	ELISA	Tekinsen and Eken (15)
Iran	49	100	>0.017-0.1	95.3	ELISA	Movassagh (16)
Turkey	40	20	0.004-0.076	5	HPLC	Kabak and Ozbey (17)
China	153	54.9	0.006-0.16	20.3	ELISA	Zheng *et al* (18)
Brazil	75	30.7	1-4.1	NR	HPLC	Oliveira *et al* (19)
Pakistan	84	41.6	LOD-0.88	25	HPLC	Iqbal *et al* (20)
Croatia	706	NR	0.00398-0.1835	9.64	ELISA	Bilandzic *et al* (21)

NR- Not reported

## Materials and methods

*Samples*

A total of 30 UHT flavored milk samples from various brands were randomly collected from supermarkets in Karaj, Alborz province, Iran, in October 2013. All samples were transported to the laboratory refrigerator and stored at 4 ^o^C and analyzed for determining the presence of AFM_1_ before their expiry date. 

*Chemicals and regents*

AFM_1_ standard was obtained from Sigma Chemical Co. Aflatoxin test immunoaffinity columns were purchased from VICAM (Watertown, MA, USA). Acetonitrile HPLC grade was purchased from Merck Co (Darmstadt, Germany). The stock solution of AFM_1_ was prepared in acetonitrile at a concentration of 0.5 μg mL^-1^ and was kept at −20 ^o^C. Working standard solutions were prepared by stock standard solution diluting acetonitrile stock solution at concentrations ranging from 0.05 to 1.5 μg mL^-1^.

*Extraction procedure and clean up by Immunoaffinity Column Chromatography*

The AFM_1_ extraction procedure from milk samples was carried out according to the official method of Institute of Standards and Industrial Research of Iran (10). 60 mL from each sample was warmed at 37 ^o^C and centrifuged at 2500 rpm and the upper fat layer was removed completely. 20 mL from skimmed milk passed through an immunoaffinity column at the flow rate of 2 mL min^−1^. The column was washed with 10 mL of water and the sorbent bed was dried and the AFM_1_ bound to the antibody was eluted with 2.5 mL of acetonitrile in 2 mL min^-1^ rate. The solution was evaporated under nitrogen gas and the residue was dissolved in 1 mL of mobile phase. 20 µL aliquot was injected into the HPLC equipment. 

*Quantitative Analysis by HPLC*

The HPLC chromatograph (Waters, Milford, MA, USA) equipped with fluorescence detector (Waters,) was used to determine AFM_1_. The chromatographic separation was achieved on a reversed phase C18 column (Waters Spherisorb, 5 µm particle size, 250 mm, 4.6 mm I.D.), at an ambient temperature. The excitation and emission wavelengths were set at 360 and 440 nm, respectively. Standard solutions of AFM_1_ with concentrations of 0.05, 0.25, 0.5, 0.75, 1, 1.25, 1.5 μg mL ^-1^ in acetonitrile were used to obtain the calibration curve. 

Acetonitrile/methanol/water (ratio 2:2:4 v/v) was used as the mobile phase at the flow rate of 1 mL/min. AFM_1_ peak in the chromatogram was identified by comparing its retention time with that of the analyzed AFM_1_ standard under the same conditions. The peak was quantified from the area under the curve of sample chromatogram by using the equation of calibration curve (*y* = 401810 +33125, *R*^2 ^= 0.996). The retention time for AFM_1 _was 5.8 min. The limits of detection and quantitation were 0.015 and 0.018 μg/L, respectively.

Recovery test was performed by spiking aflatoxin into milk samples (0.05 µg/L). Mean recovery rates was 83.3% and relative standard deviations was equal to 1.8%.

*Statistical analysis*

All statistical analyses were performed using SPSS software (Version 16; SPSS Inc., Chicago, IL) and the data were expressed as Mean ± Standard deviation (SD).

## Results and Discussion

The occurrence and the distributions levels of AFM_1 _concentration in UHT flavoured milk samples are presented in Table 1. The concentration of AFM_1_ in all samples was lower than the ISIRI and FDA limit of 0.5 μg/L (11-12). But, 10 (33.3%) of UHT flavored milk samples contained higher levels than maximum limit of 0.05 µg/L of the European Union (13).

Among aflatoxins, AFM_1_ is the most important for health for many consumers especially for children. Several surveys of aflatoxin M_1_ contamination in raw milk and milk products are reported in the other studies (1). As can be seen in Table 2. several researchers in Iran and other countries (8,9,14-21) reported the incidence of AFM_1_ contamination only in UHT milk but not the flavored milk. Our findings showed a high incidence of AFM_1_ in UHT flavoured milk samples. Kabak and Ozbey (17) reported less AFM_1_ (20%) at detectable levels in UHT milk samples; but in a recent study, Temamogullari and Kanici (22) determined AFM_1_ in 100% of the surveyed UHT milk samples from Turkey. In previous studies conducted in Iran by ELISA method, Movassagh Ghazani (23) showed that 100% (100 of 100) UHT milk samples were contaminated with AFM_1_ in Tabriz and 66.66% had levels above the EU limit. A study from India Siddappa *et al.* (24) reported AFM_1_ in UHT milk in 29 out of 45 samples (64.4%), ranging from 0.06 to 0.7 µg/L and within the positive samples 29 (100% of the total) were above the EU limit. In a previous survey by Shundo *et al.* (25) from Brazil by HPLC method, reported that 40 (100%) samples of 40 UHT milk samples had higher concentration of AFM_1_ and one sample was higher than the EU maximum limit of 0.05 µg/L, which is similar to our results. Furthermore, in Brazil, 42 UHT milk samples were screened for aflatoxin contamination and 34 (80.9%) samples contained AFM_1_. The aflatoxin of UHT milk was in the range of 0.02–0.26 µg/L (26). In another study conducted in Turkey by Aydemir Atasever *et al.* (27) identified UHT milk samples that were contaminated with aflatoxin M_1_. On the other hand, they were reported that 150 UHT milk samples collected in Erzurum, 59% (89 samples) were positive for aflatoxin M_1_ at levels of 0.05-0.185 µg/L, also 16 (10.7%) samples exceeded the maximum tolerable limit of the European Community and the Turkish Food Codex. In the present study, there was a high incidence by 33.3% of analysed raw milk samples exceed the maximum limit for AFM_1_ (0.05 µg/L) set by EU regulations and also none of samples was not above ISIRI that is similar previous studies in Brazilian by Shundo and Sabini (26), Shundo *et al.* (25) and Silva *et al.* (28). The previous study by Santini *et al.* (29) from Sicily-Italy showed that 5 (41.7%) UHT milk samples was contaminated with aflatoxin M_1_ and also none of samples was not to above 0.05 µg/L according to EU, but our results was higher than this result.

## Conclusion

This study was the first to report AFM_1_ contamination in UHT flavoured milk samples in Iran. Although none of the samples did not exceed the ISIRI limit (0.5 µg/L) but all of them were contaminated with AFM_1_. Hence, this is a warning for governmental agencies to inform farmers and dairy factories about health consequences of aflatoxins. However, concerns about aflatoxin M_1 _in milk and dairy products are common for consumers of all age groups especially for infants and children because they are the largest consumers. 
